# Characterization of a Periplasmic D-Malate:Cytochrome *c* Oxidoreductase from *Ectopseudomonas oleovorans* CECT 5344 and Its Role in Extracytoplasmic Respiration and Cyanide Detoxification

**DOI:** 10.3390/ijms26146575

**Published:** 2025-07-08

**Authors:** Faustino Merchán, Ana G. Población, María Isabel Guijo, Mar Gómez-Ortega, Felipe Morales-Durán, Irene Alonso-Ríos, Rubén Sánchez-Clemente, Rafael Blasco

**Affiliations:** Department of Biochemistry, School of Veterinary Sciences, University of Extremadura, 10003 Cáceres, Spainmguijo@unex.es (M.I.G.); margo@unex.es (M.G.-O.); felipemd@unex.es (F.M.-D.); irenear@unex.es (I.A.-R.);

**Keywords:** D-malate:cytochrome *c* oxidoreductase, cyanide, bioremediation

## Abstract

A periplasmic D-malate:cytochrome *c* oxidoreductase (DMCO) was identified in *Ectopseudomonas oleovorans* CECT5344, utilizing 2-(4-iodophenyl)-3-(4-nitrophenyl)-5-phenyl tetrazolium chloride (INT) as an artificial electron acceptor. The assay was adapted for a spectrophotometric or native polyacrylamide gel electrophoresis (PAGE) analysis. The DMCO-encoding gene (BN5_4044) was cloned and expressed in *Escherichia coli*, enabling a partial purification and biochemical characterization. In addition to D-malate, the enzyme oxidizes D-2-hydroxyglutarate and, to a lesser extent, D-lactate, with cytochrome *c* also serving as an electron acceptor. DMCO requires Zn^2+^ for activity and exists as a dimer, as determined by gel filtration. The in vitro reconstitution of the electron transfer from D-malate to oxygen was achieved using spheroplasts, enriched periplasmic fractions, and cytochrome *c*. This extracytoplasmic respiration, unique among homologs of this protein, may eliminate the need for a dedicated inner membrane transporter, thereby avoiding potential upstream respiratory bottlenecks. In the context of bioremediation, and particularly regarding the cyanide metabolism, this D-malate oxidation to oxaloacetate facilitates detoxification by forming the corresponding cyanohydrin, which can be subsequently assimilated for growth.

## 1. Introduction

L-malate is a key intermediate in the cellular metabolism, notably in the tricarboxylic acid (TCA) cycle, where it is formed by fumarase (EC 4.2.1.2) through fumarate hydration under aerobic conditions. It is subsequently oxidized to oxaloacetate by L-malate dehydrogenase (EC 1.1.1.37) or L-malate:quinone oxidoreductase (EC 1.1.5.4) or to pyruvate by malic enzymes via oxidative decarboxylation coupled with pyridine nucleotide reduction. In contrast, D-malate, typically considered a non-natural stereoisomer, is not a substrate for these enzymes. However, D-malate occurs naturally as an intermediate in aromatic compound degradation by certain *Pseudomonas* species [1,2,3]. Some bacteria, including *Escherichia coli*, utilize D-malate as a carbon and energy source through an inducible D-malic enzyme [D-malate:NAD^+^ oxidoreductase (decarboxylating), EC 1.1.1.83] [4]. In *Rhodobacter capsulatus* E1F1, D-malate assimilation is enabled by a Mn^2+^-dependent racemase [5]. Additionally, recent studies report that D-2-hydroxyglutarate dehydrogenase (D2HGDH) in *Pseudomonas stutzeri* oxidizes D-malate to oxaloacetate, potentially functioning as a D-malate oxidase and linking the D-malate metabolism to L-serine biosynthesis [6]. This metabolic versatility, observed in select species [7,8,9,10], enables *Pseudomonas* to exploit diverse carbon sources, such as D-malate, in environments like contaminated soils. This capability has significant ecological and biotechnological implications, including the bioremediation of aromatic compounds and the production of valuable chemicals.

In this study, we identified D-malate:cytochrome *c* oxidoreductase (DMCO) activity in *Ectopseudomonas oleovorans* CECT 5344 (previously *Pseudomonas pseudoalcaligenes* CECT5344 and *Pseudomonas oleovorans* CECT 5344). A novel spectrophotometric assay was developed to measure the DMCO activity using INT as an artificial electron acceptor. Cytochrome *c* was confirmed as the physiological substrate, exhibiting a Km two orders of magnitude lower than that for INT, indicating its greater efficiency as an oxidant. The enzyme’s periplasmic localization suggests the extracytoplasmic oxidation of D-malate and other D-2-hydroxy acids, enabling the energy extraction from these metabolites without specific transporters. This process converts potentially toxic D-hydroxy acids into keto acids, bypassing upstream blockages in the electron transport chain. Furthermore, keto acid formation protects this cyanotrophic bacterium from cyanide by facilitating cyanohydrin production, which is further metabolized by this strain. These findings highlight DMCO’s role in the periplasmic respiratory chain, contributing to the cyanide detoxification and biomass production from a toxic compound.

## 2. Results

### 2.1. Identification and Subcellular Localization of the D-Malate:Cytochrome c Oxidoreductase Activity

*Ectopseudomonas oleovorans* CECT5344 (R1) can assimilate cyanide as a sole nitrogen source when supplemented with an appropriate carbon source, with DL-malate identified as optimal [11,12]. L-malate, an intermediate in the TCA cycle and glyoxylate shunt, is converted to oxaloacetate via malate:quinone oxidoreductase (MQO) due to the absence of L-malate dehydrogenase in this strain [12]. Although D-malate is also utilized, its metabolic pathway remains unclear. To investigate, we measured the D-malate oxidation coupled to the reduction of 2-(4-iodophenyl)-3-(4-nitrophenyl)-5-phenyltetrazolium chloride (INT), an artificial electron acceptor that forms a detectable violet formazan precipitate [13]. Cell-free extracts of *E. oleovorans* CECT5344 catalyzed the INT reduction by D-malate, with the activity confirmed after the native polyacrylamide gel electrophoresis (see below). Previous studies suggested a periplasmic localization of this D-malate:cytochrome *c* oxidoreductase (DMCO) activity, prompting further exploration of its role in the electron transport chain. Subcellular fractionation, as detailed in Materials and Methods, was performed to assess its distribution.

**Figure 1:**
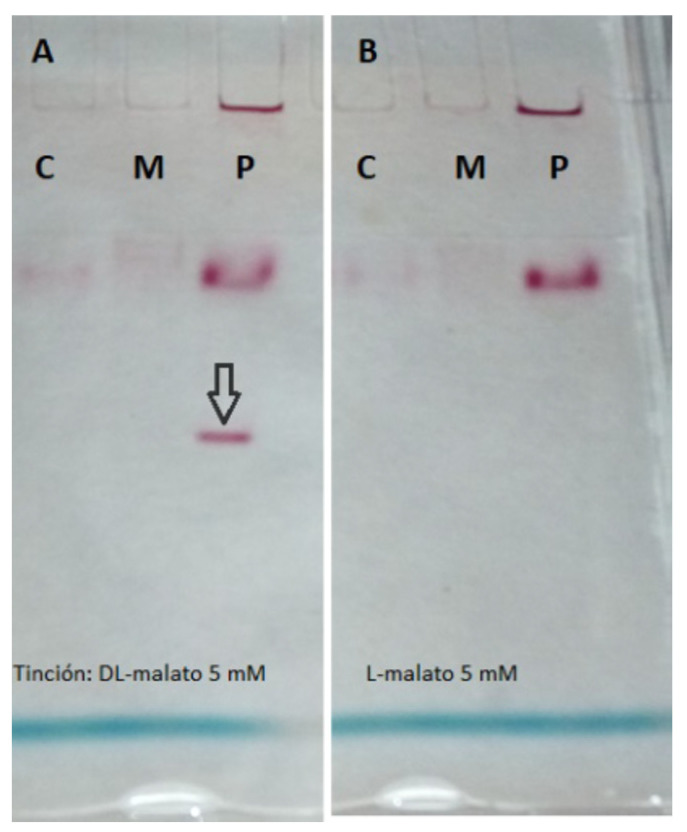
Subcellular location of the DMCO activity. Cells were grown with a commercial racemic mixture of D and L-malate as the sole carbon and energy source. After the subcellular fractionation and the subsequent native PAGE, the enzymatic assays were performed as described in the Section 4. (**A**), D-malate as a reductant. (**B**), L-malate as a reductant. The arrow indicates the position of the D-Malate:cytochrome *c* oxidoreductase activity. C: Cytoplasm. M: Membranes. P: Periplasm.

After the subcellular fractionation, specific activities of marker enzymes were measured to verify the fraction purity (Table 1): the aconitase (cytosolic), periplasmic nitrate reductase, succinate oxidase (membrane complex), and D-malate:cytochrome *c* oxidoreductase (DMCO) across all fractions. The DMCO activity was predominantly detected in the periplasmic fraction, consistently exceeding 80%. Moreover, the location of the DMCO was further confirmed by native PAGE (Figure 1).

### 2.2. The Determination of the Reaction Product of D-Malate:Cytochrome c Oxidoreductase

**Figure 2:**
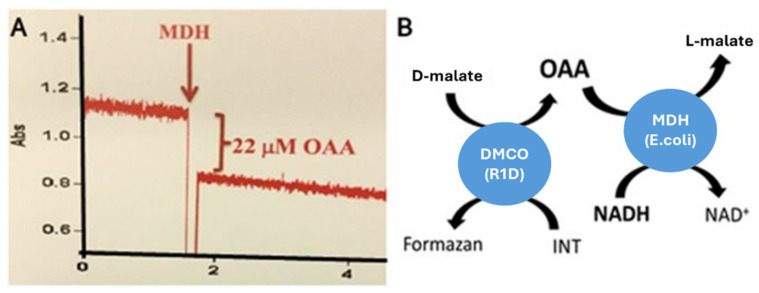
Formation of oxaloacetate (OAA) as a product of D-malate oxidation, catalyzed by DMCO. (**A**). The reaction coupled to the disappearance of NADH via the malate dehydrogenase activity. The first reaction was performed using partially purified DMCO protein, with 2 mM D-malate as the substrate and 0.2 mM INT as the electron acceptor. Approximately 25 µM of oxaloacetate was produced, which was subsequently utilized by malate dehydrogenase (MDH). Upon the addition of NADH as a cofactor, the disappearance of NADH was observed, evidenced by a decrease in absorbance measured at 340 nm (from 0.967 to 0.835). This reduction in absorbance is proportional to the oxaloacetate generated by the DMCO reaction. (**B**). A schematic representation of the D-malate oxidation catalyzed by DMCO coupled to the NADH consumption. The scheme illustrates the oxidation of D-malate catalyzed by DMCO, coupled to the consumption of NADH produced by the MDH-catalyzed oxidation of oxaloacetate to L-malate. MDH: Malate Dehydrogenase; DMCO: D-Malate:cytochrome *c* oxidoreductase; and OAA: Oxaloacetate.

To elucidate the reaction product of D-malate:cytochrome *c* oxidoreductase (DMCO) in *Ectopseudomonas oleovorans* CECT5344, its catalytic activity was coupled with L-malate dehydrogenase (MDH), as described in the Materials and Methods. L-malate oxidation by DMCO yields either oxaloacetate or pyruvate. As shown in Figure 2, the DMCO reaction product served as a substrate for commercial MDH, confirming oxaloacetate as the product of the DMCO activity.

D-malate + INT → Oxaloacetate + Formazan

### 2.3. Spectrophotometric Determination of DMCO Activity

The maximum absorption wavelength and molar extinction coefficient of the reduced INT were determined as described in the Materials and Methods. DMCO was partially purified by ammonium sulfate precipitation (40–60% saturation), followed by gel filtration chromatography on a Superdex 200 10/300 GL column (GE Healthcare), calibrated as previously reported [14]. The native enzyme’s molecular mass was estimated at ~102 kDa, suggesting a homodimeric structure. The optimal DMCO activity occurred at 45 °C, which is consistent with other enzymatic activities in *Ectopseudomonas oleovorans* CECT5 344 [14,15], and at pH 9, reflecting the bacterium’s alkalophilic nature. Under optimized conditions, the Michaelis constants (K_m_) for INT and D-malate were 3.58 ± 0.88 mM and 0.67 ± 0.07 mM, respectively.

Given that homologous DMCO proteins are Zn^2+^-dependent flavoproteins [6,16], the role of Zn^2+^ was investigated. The DMCO activity in cell-free extracts was enhanced by 2 mM ZnCl_2_ but abolished by 1 mM EDTA. Activity was restored upon the Zn^2+^ addition, confirming its requirement for catalysis, whereas other divalent cations (Ca^2+^, Mg^2+^, Mn^2+^, Cu^2+^, Fe^2+^, and Co^2+^) had no effect. Using an optimized spectrophotometric assay, the DMCO substrate specificity was determined. The high sequence identity among DMCO homologs suggests conserved conformational determinants and functional domains critical for enzymatic activity [17,18].

**Figure 3:**
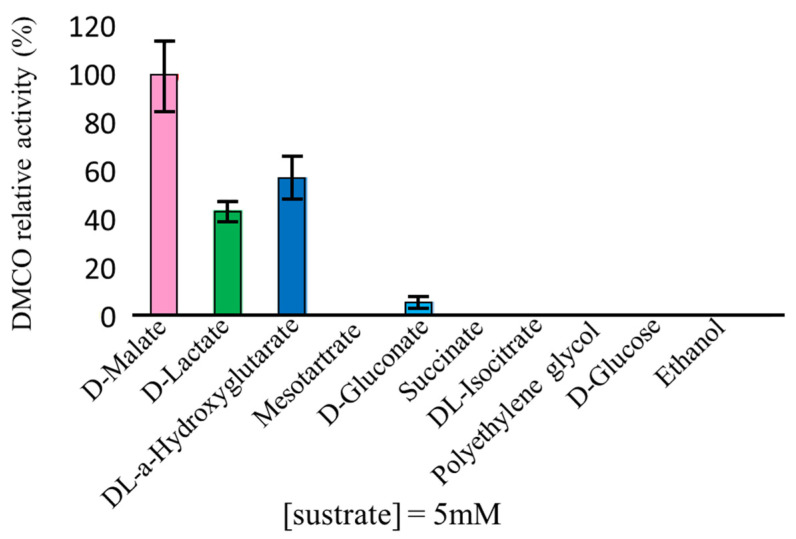
Substrate specificity of DMCO. Various compounds were used as electron donors at a final concentration of 5 mM. The highest specific activity was detected with D-malate (28.30 U/mg). Error bars represent the standard deviation of the results obtained from 4 independent assays.

The enzymatic activity of the D-malate:cytochrome *c* oxidoreductase (DMCO) was assessed using various substrates. As shown in Figure 3, DMCO exhibited the highest specific activity with D-malate (28.30 U/mg), followed by DL-2-hydroxyglutarate (16.39 U/mg), D-lactate (12.36 U/mg), and D-gluconate (1.58 U/mg). However, the Michaelis constant (K_m_) values revealed a higher affinity for DL-2-hydroxyglutarate (0.015 ± 0.004 mM) compared to D-malate (0.66 ± 0.07 mM), which is consistent with the catalytic properties of homologous enzymes in other *Pseudomonaceae* [6,18].

Using the D-2-hydroxyglutarate dehydrogenase (D2HGDH) sequence from *Pseudomonas stutzeri* A1501 (GenBank: CP000304.1) as a reference, we identified a homologous gene (BN5_4044) in the *E. oleovorans* CECT 5344 genome, sharing a 90% amino acid sequence identity. Although periplasmic localization typically requires a leader peptide [19], some proteins are exported via non-classical secretion pathways without a recognizable leader sequence [20,21,22]. Such proteins, often termed “moonlighting” proteins, exhibit dual functions in intra- and extracellular compartments [23,24]. Applying the SecretomeP-2.0 method [25], which uses artificial neural networks to predict non-classical secretion based on protein characteristics, DMCO yielded a score of 0.518 (threshold ≥ 0.5 for bacterial sequences), supporting its experimentally determined periplasmic localization without a canonical leader sequence.

The DMCO gene in *E. oleovorans* CECT 5344 is located downstream of *serA*, which encodes D-3-phosphoglycerate dehydrogenase, a synteny conserved across multiple *Pseudomonas* species. This genomic arrangement, combined with experimental evidence, supports the hypothesis that DMCO (as a D2HGDH homolog) converts D-2-hydroxyglutarate to 2-ketoglutarate, facilitating L-serine biosynthesis by overcoming the thermodynamic barrier of the D-3-phosphoglycerate (D-3-PG) dehydrogenation to 3-phosphohydroxypyruvate (3-PHP) catalyzed by SerA [26]. However, the predominant periplasmic localization of DMCO complicates its role in cytoplasmic L-serine biosynthesis [6,27].

**Figure 4:**
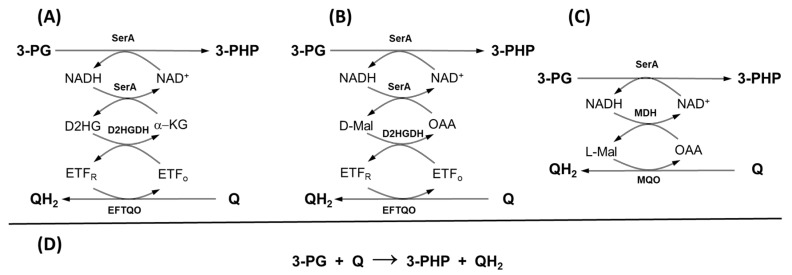
Breaking the thermodynamic barrier of the reaction catalyzed by SerA. In all cases the electrons are transferred from 3-phosphoglycerate (3-PG) to the quinone pool (Q), (general reaction (**D**)). The electron transfer flavoprotein (ETF) may drive the electrons to the quinone pool either with D2HG (**A**) or D-malate (**B**) as an intermediate. The same reactions could be hypothetically catalyzed by the consecutive action of serine dehydrogenase (SerA), L-malate dehydrogenase (MDH), and L-malate:quinone oxidoreductase (MQO) (**C**).

Notably, both D-2-hydroxyglutarate (Figure 4A) and D-malate (Figure 4B) can alleviate the thermodynamic constraint of the SerA activity by consuming NADH and donating electrons to the electron transport chain (ETC). Similarly, L-malate can serve as an electron donor either through the MQO [28] (Figure 4C) or when NAD-dependent malate dehydrogenase is active, as observed in *Escherichia coli* [29], *Corynebacterium* [30], and some *Pseudomonas* species [31].

### 2.4. Mutagenesis of DMCO in Ectopseudomonas oleovorans CECT 5344 R1D

**Figure 5:**
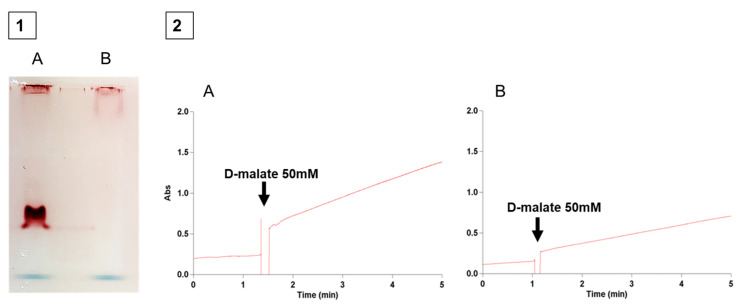
The DMO oxidase of the DMO^−^ mutant strain. The D-malate oxidase activity detected in native electrophoresis in polyacrylamide gels using INT as an electron acceptor and D-malate as an electron donor, (**1**) or spectrophotometrically (**2**). The cell-free extract of the strain Ectopseudomonas oleovorans CECT 5344 R1D wt (**A**) or the DMO^−^ mutant strain (**B**).

A knockout mutant of the DMCO gene (BN5_4044) in *Ectopseudomonas oleovorans* CECT 5344 R1D was generated via a double homologous recombination, as described in the Materials and Methods. The DMCO^−^ mutant exhibited no D-malate dehydrogenase activity, as confirmed by polyacrylamide gel electrophoresis (PAGE; Figure 5(1)) and spectrophotometric assays (Figure 5(2)).

The spectrophotometric assay demonstrated greater quantitative sensitivity and precision compared to the polyacrylamide gel method (Figure 5). It effectively distinguished between the non-specific chemical reduction of INT by cell-free extract components and the enzymatic reduction catalyzed by DMCO (Figure 5).

#### TPhenotype of the Mutant with Different Carbon Sources and the Influence of Cyanide

**Figure 6:**
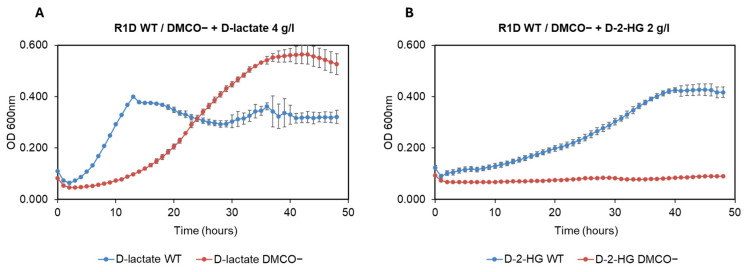
Growth curves on D-lactate (**A**) and D-2-HG (**B**) of the CECT 5344 wild-type and DMCO^−^ mutant strains.

**Figure 7:**
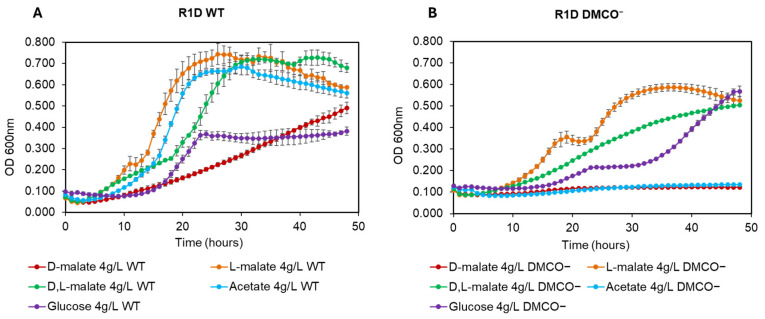
Growth curves with different carbon sources of the wild-type (**A**) and DMCO mutant (**B**).

The wild-type *Ectopseudomonas oleovorans* CECT 5344 strain utilized D-lactate and D-2-hydroxyglutarate (D-2-HG) as carbon and energy sources for growth (Figure 6). A DMCO mutant strain failed to metabolize D-2-HG (Figure 6B) and D-malate (Figure 7B), indicating that DMCO is the sole enzyme responsible for their oxidation. However, the DMCO mutation did not impair the growth on D-lactate (Figure 6A), suggesting the presence of an alternative D-lactate oxidation pathway.

In addition to D-malate, the DMCO^−^ mutant was unable to use acetate as a carbon and energy source (Figure 7). The cell growth of the mutant with D, L-malate was significantly lower than the wild-type (Figure 7). Moreover, the mutant showed a diauxic growth curve with glucose and L-malate as carbon sources.

**Figure 8:**
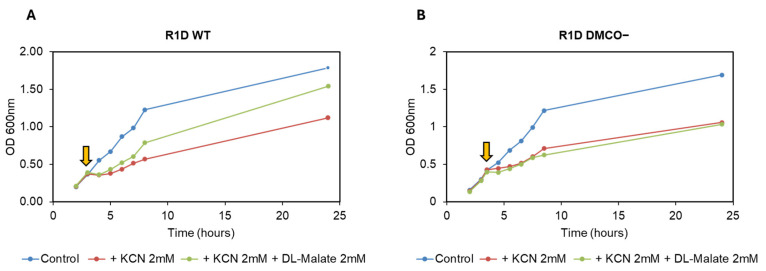
Effect of cyanide on the cellular growth of the wild-type (**A**) and DMCO^−^ mutant strain of CECT 5344 (**B**) in the LB medium, in the presence of D-malate. Arrows indicate the time point when KCN 2 mM was added to the cultures.

*Ectopseudomonas oleovorans* CECT 5344 has been extensively studied for its potential in cyanide bioremediation due to its resistance to cyanide and its ability to utilize it as a nitrogen source under alkaline pH conditions, which minimizes the HCN volatilization and environmental contamination [11]. Effective cyanide assimilation requires a suitable carbon source, with DL-malate identified as optimal [12]. As L-malate is converted to oxaloacetate—which reacts with cyanide to form cyanohydrin, potentially reducing cyanide toxicity [28]—we investigated the role of D-malate oxidoreductase (DMCO) in mitigating cyanide’s toxic effects. The cyanide addition to LB-grown cells was toxic to both wild-type and DMCO-deficient mutant strains; however, D-malate conferred a protective effect only in the wild-type strain (Figure 8).

**Figure 9:**
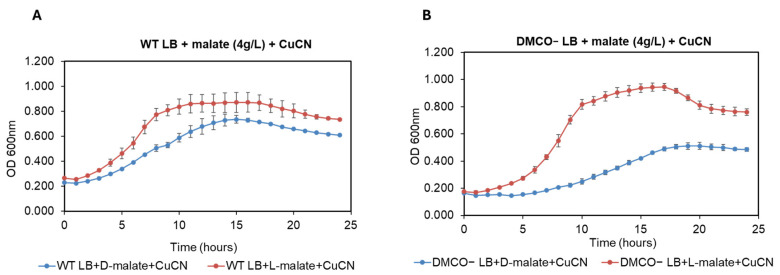
Effect of D- or L-Malate as protective agents vs. cyanide (0.5 mM Cu(CN)_4_^2−^) in LB-growing cells of the wild-type and DMCO mutant of CECT5344 strains. The wild-type (**A**) and DMCO mutant of the CECT 5344 strain (**B**) were inoculated in the LB-medium in the presence of 4 g/L of L-malate (red) or D-malate (blue). The absorbance at 600 nm was recorded at the indicated times.

Since cyanide slowly evaporates, even at an alkaline pH, the experiments were performed in the presence of a 0.5 mM Cu (CN)_4_^2−^ complex to compare the putative protective effect of D- and L-malate (Figure 9). Indeed, L-malate was protected from the cyanide toxicity in both the wild-type and the DMCO^−^ mutant, whereas the effect of D-malate was only significant in the case of the wild-type (Figure 9).

### 2.5. The Determination of Cytochrome c as the Physiological Substrate for DMCO

**Figure 10:**
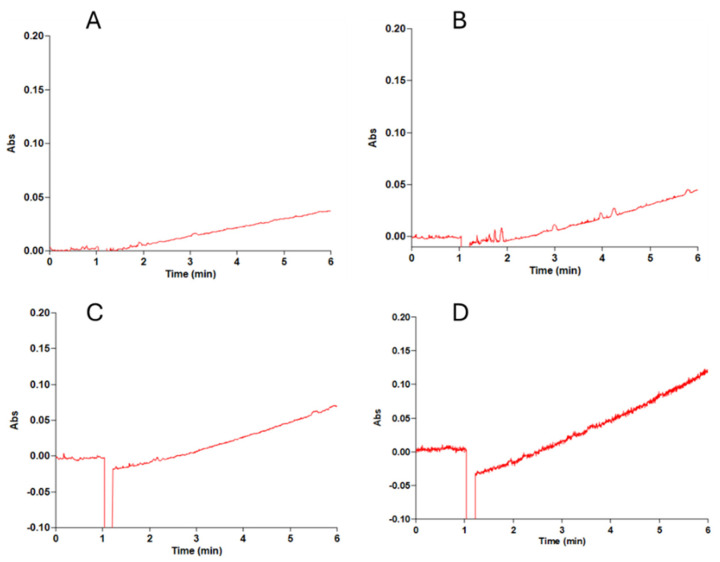
The DMCO activity with cytochrome *c* as an electron acceptor. The reaction mixture (final volume: 1 mL) contained 50 mM Tris-HCl buffer (pH 9.0), 2 mM ZnCl_2_, and 20 μL of partially purified DMCO enzyme (60 U/mL). The mixture was equilibrated at 45 °C for approximately 1 min before initiating the reaction by adding varying concentrations of cytochrome *c*. The reaction was monitored at 550 nm. The slope of the absorbance curve was determined between 2 and 5 min, during the linear phase of the reaction. (**A**): [cytochrome c] = 20 μM, (**B**): [cytochrome c] = 50 μM, (**C**): [cytochrome c] = 100 μM, and (**D**): [cytochrome c] = 200 μM.

Given the predominant periplasmic localization of D-malate:cytochrome *c* oxidoreductase (DMCO) in *Ectopseudomonas oleovorans* CECT 5344, its homology to bacterial DMCO proteins using cytochrome *c* as a substrate [26], and the analogous localization of mitochondrial D-lactate dehydrogenase in the intermembrane space of *Arabidopsis thaliana* [32], we investigated DMCO’s role in the electron transport chain. The DMCO activity was measured spectrophotometrically by monitoring the increase in the absorbance at 550 nm (α-band of reduced cytochrome *c*, ε_550nm_ = 21.2 mM^−1^ cm^−1^). The assay temperature and composition of the reaction mixture composition were identical to those used in the spectrophotometric assay (see Section 4), except that cytochrome *c* was used in place of INT. The activity increased by increasing cytochrome *c* concentrations (Figure 10), and the Km was determined to be 65.1 ± 10 μM, which is consistent with homologous enzymes [26].

**Figure 11:**
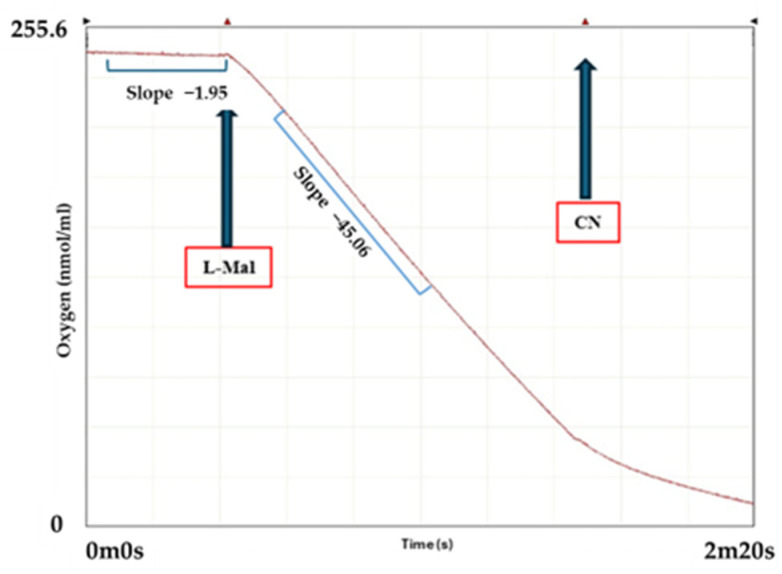
Functional integrity of the purified membrane fraction of the CECT 5344 strain. The oxygen consumption was measured by adding L-malate as a substrate to the purified membranes fraction of the CECT5344 strain (400 μg protein). The arrows indicate the addition of L-Mal: L-Malate (5 mM) and CN: Potassium cyanide (2 mM).

To confirm cytochrome *c* as an electron acceptor for the D-malate:cytochrome *c* oxidoreductase (DMCO) activity, we reconstituted the in vivo electron transport from D-malate to oxygen in vitro. The purified membrane fraction’s activity was validated using a Clark-type oximeter, demonstrating the L-malate oxidation by oxygen (Figure 11), which is consistent with its role as a direct electron donor to the electron transport chain via L-malate:quinone oxidoreductase in *Ectopseudomonas oleovorans* CECT 5344 [12,28]. The potassium cyanide addition inhibited the oxygen consumption, further confirming the integrity of the electron transport chain.

**Figure 12:**
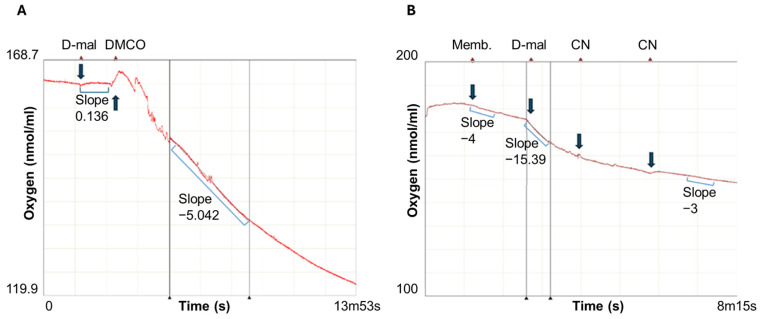
Reconstruction of the physiological DMCO activity in the CECT5344 strain. To determine whether D-malate oxidase gives up the electrons from D-malate to the electron transport chain, the following assay was carried out on a Clark-type oxygen oximeter: (**A**): the oxygen consumption was measured in a 1 mL reaction mixture containing a 50 mM Tris-HCl pH 9 buffer and 20 μL of a purified membrane fraction (400 μg protein). At the times indicated by an arrow, 5 mM D-malate (D-Mal) and 100 μL of partially purified DMCO (DMCO (65 mg protein)) were added. (**B**): starting with 100 μL of periplasmic fraction and adding 20 μL purified membrane fraction. Reaction was triggered with 5 mM D-Malate (D-Mal) and including Zn^2+^ 2 mM.

By contrast, D-malate was not a substrate unless a fraction containing the DMCO activity was added (Figure 12A).

The addition of the 2 mM Zn^2+^ in the coupled assay significantly increased the oxygen consumption (Figure 12). DMCO exhibited a specific activity of 77.5 mU/mg in the coupled assay with Zn^2+^ (Figure 12B) and 92.5 mU/mg when measured spectrophotometrically with INT. These results confirm that DMCO, a metalloprotein, mediates the electron transfer from D-malate to the electron transport chain, ultimately reducing the molecular oxygen via cytochrome *c*.

## 3. Discussion

The genus *Pseudomonas* exhibits a remarkable nutritional versatility [33,34]. The *Ectopseudomonas oleovorans* CECT5344 strain, with its diverse genomic capabilities, can utilize various substrates, including cyanide-derived compounds [35], which are significant environmental pollutants requiring complex and costly remediation [36]. This strain efficiently assimilates cyanide as a nitrogen source, demonstrating a high resistance and dependence on a carbon source, with malate being particularly effective [11,12]. Enzymatic studies revealed a periplasmic D-malate:cytochrome *c* oxidoreductase (DMCO) activity that oxidizes D-malate to oxaloacetate, independent of NADH, using INT as an artificial electron acceptor. This activity was initially detected via native polyacrylamide gel electrophoresis with INT staining, producing a reddish formazan precipitate (Figure 1) [13]. To improve efficiency, a spectrophotometric assay was developed, enabling the rapid and sensitive detection of the DMCO activity and its biochemical characterization.

The DMCO enzyme, localized in the periplasm, also oxidizes D-2-hydroxyglutarate (2-DHG). Its gene was identified in CECT 5344 by the homology to D-2-hydroxyglutarate dehydrogenases in *Pseudomonas stutzeri* and *P. aeruginosa* PAO1, sharing a 90% protein identity with the *P. stutzeri* ortholog [6,16,18]. Both enzymes require Zn^2+^ and FAD as cofactors, but CECT5344’s DMCO exhibits a distinct substrate specificity, with activity on D-lactate and D-gluconate and significantly lower K_m_ values for 2-DHG (0.015 ± 0.004 mM vs. 0.17 ± 0.02 mM) and D-malate (0.66 ± 0.07 mM vs. 3.61 ± 0.14 mM), indicating over a fivefold higher catalytic efficiency for D-malate [6].

In *P. stutzeri*, DMCO is proposed to facilitate L-serine synthesis by overcoming the thermodynamic barrier of D-3-phosphoglycerate dehydrogenation [6]. While this role is plausible in CECT 5344, the enzyme’s periplasmic localization, enhanced substrate specificity, and greater efficiency suggest an alternative function. Other mechanisms may also bypass this thermodynamic barrier (Figure 4).

This study identifies cytochrome *c* as the primary electron acceptor for D-malate:cytochrome *c* oxidoreductase (DMCO, BN5_4044) in *Ectopseudomonas oleovorans* CECT 5344, which is analogous to plant mitochondrial D-lactate dehydrogenase [32], despite no sequence similarity with a potential D-lactate dehydrogenase (BN5_0898). DMCO facilitates the electron transfer from D-malate to cytochrome *c*, integrating into the electron transport chain (ETC) to support energy production.

*E. oleovorans* CECT 5344 exhibits metabolic versatility, utilizing carbon sources such as D,L-malate, D-lactate, acetate, and succinate via the tricarboxylic acid (TCA) cycle and glyoxylate shunt [28,37,38]. DMCO oxidizes D-malate to oxaloacetate (OAA), fueling the TCA cycle and biosynthesis in related *Pseudomonas* species [1]. Acetate is metabolized through the glyoxylate shunt, producing malate and OAA while bypassing TCA decarboxylation [37,38]. Additionally, a malate:quinone oxidoreductase (MQO) generates OAA, which detoxifies cyanide by forming cyanohydrins, enhancing the survival in toxic environments [28].

The currently accepted pathway for the cyanide assimilation in the CECT 5344 strain involves the nitrilase NitC, which is proposed to catalyze the hydrolysis of oxaloacetate cyanohydrin, thereby releasing ammonium—a readily assimilable nitrogen source [39,40]. However, to the best of our knowledge, this enzymatic activity has never been demonstrated in vitro. In addition to ammonium, the reaction would also produce a tricarboxylic compound which, unless proven otherwise, is likely a metabolic dead-end. More precisely, the reaction between cyanide and oxaloacetate is expected to yield a mixture of racemic cyanohydrins, resulting in a pool of potentially non-metabolizable compounds. Given these uncertainties, we argue that the cyanohydrin pathway does not represent a credible route for cyanide assimilation. Instead, we propose that cyanohydrin formation serves as a defensive mechanism to reduce the concentration of free cyanide. Since cyanohydrin formation is a reversible process, the assimilation of free cyanide via yet unidentified metabolic pathways could shift the equilibrium, regenerating oxaloacetate—a tricarboxylic acid (TCA) cycle intermediate that may serve as a source of carbon and energy. 

A DMCO-deficient mutant (DMCO^−^) failed to utilize D-malate and D-2-hydroxyglutarate as carbon sources but retained the D-lactate metabolism (Figure 6 and Figure 7). In the LB medium supplemented with D-malate, the wild-type strain grew robustly by oxidizing D-malate to OAA via DMCO, supporting the TCA cycle flux [1]. The DMCO^−^ mutant exhibited impaired growth, likely due to the following: (a) The competition for carboxylate transport: D-malate accumulation in the mutant may saturate shared transporters (e.g., DctA [41]), limiting the uptake of other carboxylates and reducing the metabolic flux; (b) Enzymatic inhibition: Accumulated D-malate may inhibit TCA cycle enzymes (e.g., malate dehydrogenase, fumarase [42,43]), disrupting OAA production and biosynthesis; and (c) Redox imbalance: The loss of DMCO-mediated D-malate oxidation reduces NADH production, altering NADH/NAD^+^ ratios and impairing ETC efficiency and ATP synthesis [44,45]. These effects highlight DMCO’s role in efficient growth, even in nutrient-rich media.

On acetate, the wild-type strain employs the glyoxylate cycle to convert acetyl-CoA into malate and OAA for biomass synthesis [43]. The DMCO^−^ mutant’s growth defect suggests additional roles for DMCO beyond D-malate metabolism, potentially due to the following: (a) Redox perturbations: D-malate accumulation may elevate NADH/NAD^+^ ratios, inhibiting TCA and glyoxylate cycle enzymes (e.g., isocitrate dehydrogenase) and reducing the ETC efficiency and ATP production [44,45,46]. This aligns with observations of redox-sensitive growth deficits in the acetate metabolism [47]. (b) The interference with alternative pathways: Excess D-malate may inhibit MQO or other enzymes, disrupting the carbon flux and precursor availability [42,48] and reducing metabolic flexibility, a key trait in *Pseudomonas* [49]. (c) Metabolic shifts: The blocked D-malate metabolism may redirect carbon to less efficient pathways, lowering energy and biomass yields [47,48]. These findings underscore DMCO’s regulatory role in acetate utilization and metabolic flexibility in *Pseudomonas*.

The primary distinction of this study from prior reports lies in the periplasmic localization of D-malate oxidoreductase (DMCO) in *Ectopseudomonas oleovorans* CECT5344. This localization challenges the proposed role of this enzyme in cytoplasmic L-serine biosynthesis, particularly as a link between glycolysis intermediates (e.g., D-3-phosphoglycerate), the tricarboxylic acid cycle (e.g., 2-ketoglutarate), and L-serine biosynthesis pathways [27]. The abundance of genetic systems for assimilating diverse carbon and nitrogen sources, often toxic to other organisms [34,50], suggests a specialized physiological role for DMCO. These systems indicate that CECT 5344 likely evolved in environments rich in such compounds, including cyanide, which the strain can resist and metabolize. In cyanide-rich conditions, CECT5344 activates alternative metabolic pathways, including cyanide-insensitive terminal oxidases and iron-independent isoenzymes to counter cyanide’s metal-chelating effects [51,52]. However, cyanide assimilation imposes metabolic stress and requires active transport systems for intracellular degradation and nitrogen utilization.

The periplasmic localization of DMCO provides a survival advantage by enabling external respiration and cyanide detoxification without dedicated transport systems. DMCO oxidizes specific oxoacids, donating electrons to cytochrome *c*, which supports an alternative respiratory pathway in the periplasm. This activity reduces the need for extensive gene expression changes, as DMCO neutralizes cyanide before it reaches the inner membrane, preventing the inhibition of terminal oxidases and minimizing respiratory chain disruption.

**Figure 13:**
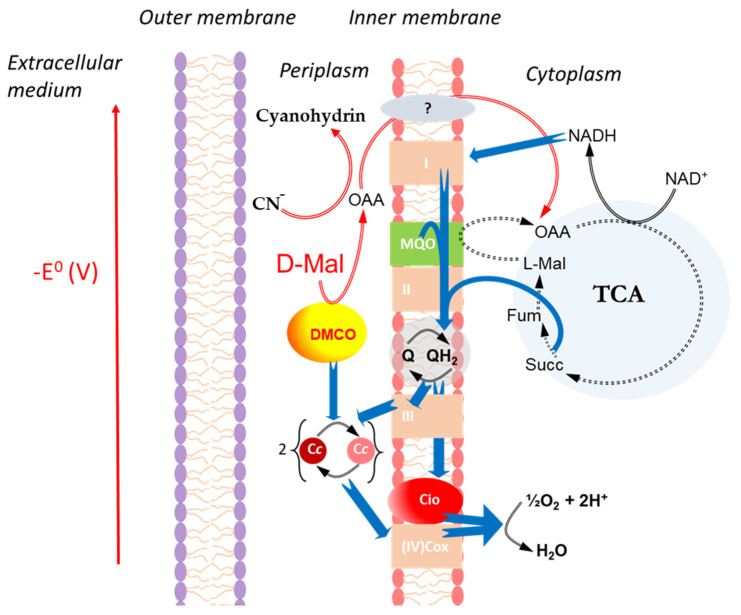
Proposed role of DMCO in the energetic metabolism of *Ectopseudomonas oleovorans* CECT 5344. D-malate oxidase (DMCO) catalyzes the electron transfer from D-malate to cytochrome *c.* The electron flow is represented by blue arrows. TCA: Tricarboxylic Acid Cycle (closed as a dotted arrow). Cio: Cyanide-Insensitive Oxidase. MQO: L-Malate: Quinone Oxidoreductase. Respiratory complexes I (NADH dehydrogenase), II (succinate dehydrogenase), III (*bc*_1_ complex), and IV (Cox-Cytochrome *c* oxidase) are represented as orange squares in the membrane. Cc: Cytochrome c (reduced—pink, oxidized—red). The vertical axis qualitatively represents the (negative) standard reducing potential from NADH to molecular oxygen.

Moreover, DMCO’s oxidation of D-malate generates oxaloacetate (OAA) in the periplasm, which reacts with cyanide to form non-toxic cyanohydrins, assimilable by the bacterium [28]. This dual role—energy production via electron transfer and cyanide detoxification—enhances the cellular resilience by reducing metabolic adaptations and the reliance on less efficient electron transport under cyanide stress. Figure 13 illustrates DMCO’s role in the periplasmic space of *E. oleovorans* CECT5344, highlighting its strategic contribution to metabolic efficiency and survival in cyanide-contaminated environments.

## 4. Materials and Methods

### 4.1. Biological Material

The subject of this study is the microorganism *Ectopseudomonas oleovorans* CECT 5344, although the strain used in the laboratory is denoted as R1, a spontaneous mutant resistant to rifampicin (50 μg/mL), which facilitates its selection.

### 4.2. Culture Media

Luria–Bertani (LB) Medium. To prepare 1 L of Luria–Bertani (LB) medium, the following components were dissolved in 1 L of deionized water: 10 g/L bactotryptone, 10 g/L sodium chloride, and 5 g/L yeast extract. pH was adjusted using 1 M NaOH to either pH 8.5 for the cultivation of *Ectopseudomonas oleovorans* CECT 5344 or pH 7.5 for *Escherichia coli*.

M9 Minimal Medium. To prepare 1 L of M9 minimal medium, the following components were dissolved in 1 L of deionized water: 6 g/L Na_2_HPO_4_, 3 g/L KH_2_PO_4_, 0.5 g/L NaCl, and 2 mL/L trace element solution. The trace element solution consisted of the following, dissolved in 1 L of deionized water: 10.75 g/L MgCl_2_, 2 g/L CaCO_3_, 6.16 g/L MgSO_4_·7H_2_O, 4.75 g/L FeSO_4_·7H_2_O, 1.44 g/L ZnSO_4_·7H_2_O, 1.12 g/L MnSO_4_·7H_2_O, 0.25 g/L CuSO_4_·5H_2_O, 0.28 g/L CoSO_4_·7H_2_O, 0.06 g/L H_3_BO_3_, and 51.3 mL/L 12 N HCl. M9 minimal medium was used to investigate bacterial growth, with the carbon source varied according to experimental requirements. Carbon sources included D,L-malate, D-malate, or L-malate, each at a concentration of 4 g/L, depending on the specific metabolic study. Ammonium chloride (NH_4_Cl) was added as the nitrogen source at a final concentration of 5 mM.

### 4.3. Determinación of Cell Growth

Growth curves were generated by measuring absorbance at a wavelength of 600 nm over time using a Multiskan FC microplate photometer (Thermo Fisher Scientific Inc. Waltham, MA, USA). A 96-well plate was designed and inoculated with the strains *Ectopseudomonas oleovorans* CECT 5344 R1D and *Ectopseudomonas oleovorans* CECT 5344-DMCO^−^. Prior to the experiment, both strains were cultured overnight in liquid Luria–Bertani (LB) medium (pH 8.5) at 30 °C. The following day, the cultures were transferred to M9 minimal medium (1×) and, after adaptation to this medium, were inoculated into the corresponding wells of the 96-well plate. In the 96-well plate, cells were cultivated in M9 medium (10×) supplemented with NH_4_Cl (1 g/L) as the nitrogen source. The carbon source was varied using different isomers of the compound of interest: D,L-malate (4 g/L), D-malate (4 g/L), and L-malate (4 g/L). Additionally, a blank and a negative control, lacking a carbon source (source-free control, SFC), were included alongside the replicates for each isomer. Three technical replicates were performed for each sample. The Multiskan FC photometer was programmed to measure absorbance under optimized bacterial growth conditions at 30 °C, with continuous measurements taken every 30 min over a total duration of 48 h. Cultures were agitated to ensure homogeneity during the assay.

The objective of these growth curve assays was to evaluate the impact of varying carbon sources (different malate isomers) on the growth dynamics of the two bacterial strains.

### 4.4. Preparation of Cell-Free Extracts

Cells (500 mL of culture) were harvested by centrifugation at 9632× *g* for 15 min at 4 °C (Avanti™ J-25, Beckman Coulter, Brea, CA, USA). The supernatant was discarded, and the pellet was washed twice with 50 mM Tris/HCl buffer containing 10% glycerol, pH 8. Finally, the cells were homogenized in 3.5 mL of Tris/HCl buffer pH 8, 10% glycerol, and protease inhibitor (F. Hoffmann-La Roche Ltd., Basel, Switzerland). The extracts were stored at −20 °C for preservation and subsequent use. The collected cells from the previous step were subjected to disruption by pressure difference using a French press at approximately 68 atm (mini-cell, Thermo Fisher Scientific Inc., Massachusetts, USA). The resulting lysate was centrifuged for 15 min at 4 °C at 12,857× *g* (Eppendorf 5810, Eppendorf, Hamburg, Germany). The supernatant obtained was ultracentrifuged in an Optima™ TLX ultracentrifuge (Beckman Coulter, California, USA, rotor TLA 110) at 70,131× *g* for 1 h at 4 °C. The supernatant was recovered and stored at −20 °C for further analysis.

### 4.5. Subcellular Fractionation

Subcellular fractionation was performed according to the protocol by Imperi et al. [53] with modifications. Cells were harvested at an OD_600_ nm ≈ 0.6 (4000× *g*, 10 min at 4 °C, rotor JA-14, Avanti™ J-25, Beckman Coulter, CA, USA) and washed twice with 30 mM Tris/HCl buffer, pH 7.1, and 150 mM NaCl. Subsequently, the bacterial pellet from one liter of culture was resuspended in 30 mL of 30 mM Tris/HCl pH 8, 20% sucrose, 4 mM EDTA, 0.5 mg/mL lysozyme, and 1 mM PMSF and incubated for 60 min in a thermostatic bath at 30 °C with gentle agitation. Two minutes after the start of incubation, MgCl_2_ was added to a final concentration of 10 mM. After the incubation period, the suspension was centrifuged (11,000× *g* for 15 min at 4 °C), and the supernatant (periplasmic fraction) was collected. Glycerol (10%) was added to this fraction as a preservative for enzymatic activities. The pellet (spheroplasts) was resuspended in 3 mL of 30 mM Tris/HCl, pH 8, and 10 mM MgCl_2_ and subjected to pressure disruption using the French press under the conditions described previously. The lysate was then centrifuged gently (15 min at 4 °C at 12,857× *g* (10,000 rpm) in an Eppendorf 5810 centrifuge, rotor F-34-6-38) to remove cellular debris and unbroken cells. The supernatant was ultracentrifuged in an Optima™ TLX ultracentrifuge (Beckman Coulter, California, USA, rotor TLA 110) at 70,131× *g* (41,000 rpm) for 1 h at 4 °C. Finally, the supernatant (cytoplasmic fraction) was carefully separated, and the pellet was homogenized in 3 mL of 30 mM Tris/HCl buffer with 10% glycerol, constituting the membrane fraction.

### 4.6. Analytical Determinations

#### 4.6.1. Determination of Cyanide Concentration

The concentration of cyanide was determined spectrophotometrically by adapting the method described by Luque et al. [11]. The procedure is as follows: 1 mL of an appropriately diluted sample (for an initial concentration of 2 mM, approximately a 250-fold dilution is necessary) and 0.040 mL of 1% (*w*/*v*) chloramine T was added and incubated at room temperature for 5 min. Then, 0.120 mL of Reagent B (3 g of barbituric acid, 15 mL of pyridine, 3 mL of HCl, and distilled water to make 50 mL) was added. The mixture was incubated for approximately 10 min, after which the absorbance at 578 nm was measured. The value obtained was interpolated on a standard curve prepared previously with known concentrations of KCN.

#### 4.6.2. Determination of Enzymatic Activities

##### Fumarase Activity: L-Malate Hydro-Lyase (EC 4.2.1.2)

Fumarase activity was determined by measuring the appearance of fumarate (ε = 2.4 mM^−1^ cm^−1^) at 50 °C, observed as an increase in absorbance at 240 nm [54]. The reaction mixture contained 50 mM Tris/HCl pH 8, 20 mM L-malate, and an appropriate amount of cell-free extract in a final volume of 1 mL.

##### Aconitase Activity (EC 4.2.1.3)

Aconitase activity was determined as described by Becerra León, G. [4], by measuring the production of the reaction intermediate, cis-aconitate (ε = 3.6 mM^−1^ cm^−1^), observed as an increase in absorbance at 240 nm at 60 °C. The reaction mixture consisted of 50 mM Tris/HCl pH 8, 30 mM isocitrate, an appropriate amount of cell-free extract, and distilled water to a final volume of 1 mL.

##### Nitrate Reductase Activity (EC 1.7.99.4)

For the determination of nitrate reductase activity, cell extracts were treated as follows: to an appropriately diluted cell-free extract, the following were added: 0.1 mL of 100 mM nitrate, 0.2 mL of 0.5 M Tris/HCl pH 8, 0.1 mL of methyl viologen, and 0.1 mL of sodium dithionite (8 mg/mL dissolved in 0.5 M Tris/HCl pH 8). The mixture was incubated at 30 °C for 10 min. The reaction was stopped by agitation, and 1 mL of sulfanilamide (20 mL of 37% HCl, 1 g of 99% sulfanilamide, and distilled water to 100 mL) was added, followed by 1 mL of N-naphthylethylenediamine (20 mg of N-NEDA made up to 100 mL with distilled water). After 10 min at room temperature, the absorbance at 540 nm was measured, and the results were extrapolated to a nitrite calibration curve. For the standard curve, known concentrations of nitrite were used, adding 1 mL of sulfanilamide and 1 mL of N-NEDA to each, allowing them to react for 10 min at room temperature and measuring the absorbance at 540 nm.

##### Succinate Dehydrogenase Activity (EC 1.3.5.1)

This activity was determined as an indicator of membrane fraction integrity by measuring oxygen consumption coupled to the addition of succinate. An oxygen electrode type Clark (Hansatech, Norfolk, United Kingdom) was used to measure oxygen consumption upon adding 10 μL of 500 mM succinate (final concentration 5 mM) to a reaction mixture consisting of 50 μL of cell-free extract, 100 μL of 0.5 M Tris/HCl pH 8, and distilled water to 1 mL.

##### Malate:Quinone Oxidoreductase Activity (EC 1.1.5.4)

This activity, like succinate dehydrogenase, was measured by coupling to the electron transport chain associated with membranes, with oxygen as the final acceptor. Using a Clark-type oxygen electrode (Hansatech, King’s Lynn, Norfolk, United Kingdom), oxygen consumption was determined in an aqueous solution containing 50 µL of 1 M Tris/HCl pH 8 (final concentration 50 mM), 10 µL of membrane extract, and distilled water to 1 mL. The reaction was initiated by adding 25 µL of 200 mM L-malate (final concentration 5 mM), and oxygen consumption was detected. To confirm that oxygen consumption was due to cellular respiration, inhibition was verified by adding 10 µL of 200 mM KCN.

##### Characterization of D-Malate Oxidase Activity (DMCO)

The reduction of INT to form the insoluble colored compound formazan has been used on several occasions to measure enzymatic activities in native polyacrylamide gels [55].

##### Native Polyacrylamide Gel Electrophoresis (Native-PAGE)

Prior to the development of the spectrophotometric method, this technique was employed to detect D-malate oxidase activity, as it allows proteins to migrate without denaturation, thus moving based on charge, size, and shape [55], while retaining their activity. The buffer system used was Tris-glycine (25 mM Tris-base, 250 mM glycine, pH 8.3). Electrophoresis was performed using a Mini-Protean^®^ 3 Cell (BioRad, California, USA) at a constant voltage of 100 V, provided by a Power Pac Basic power supply (BioRad, California, USA) and kept at 4 °C to prevent overheating and protein denaturation. The loading buffer (4×) contained 40% (*v*/*v*) glycerol, 0.004% (*w*/*v*) bromophenol blue, and 0.125 M Tris/HCl pH 6.8. The gel was developed with the following reagents: 5 mM D- or L-malate, 50 mM Tris-glycine pH 9, 2 mM INT (2-(4-iodophenyl)-3-(4-nitrophenyl)-5-phenyl-2H-tetrazolium), 2 mM ZnCl_2_, and distilled water to the final volume. The gel was gently agitated until a reddish band appeared, resulting from the action of DMCO activity (D-malate oxidase catalyzes the oxidation of DL-malate, reducing INT to formazan, which forms a reddish precipitate). The developed gel was then washed several times with distilled water and immersed in a gel preservation solution containing 10% acetic acid, 5% glycerol, and 85% distilled water for subsequent analysis.

##### Spectrophotometric Method

This activity was measured spectrophotometrically at 495 nm and 45 °C. The reaction mixture, for a final volume of 1 mL, contained 50 mM Tris-glycine buffer pH 9, 2 mM INT (2-(4-iodophenyl)-3-(4-nitrophenyl)-5-phenyl-2H-tetrazolium), 2 mM ZnCl_2_, and appropriately diluted cell-free extract or cellular fraction. The kinetics were initiated by adding 5 mM D-malate. Activity was determined by the increase in slope, considering that the calculated molar extinction coefficient of reduced INT was ε = 13.17 mM^−1^ cm^−1^.

The same method was used to measure the activity with cytochrome *c* as substrate, except that cytochrome *c* was used in place of INT. Again, in this case the activity was followed by monitoring the increase in absorbance at 550 nm (α-band of reduced cytochrome *c*, ε_550 nm_ = 21.2 mM^−1^ cm^−1^).

##### Determination of DMCO Substrate Specificity

The following reagents were used as potential electron donors for the activity: D-malate, L-malate, D-glucose, D-gluconate, ethanol, polyethylene glycol, succinate, mesotartrate, lactic acid, and D-α-hydroxyglutarate, all at a final concentration of 5 mM. To determine if the activity requires cofactors, assays were performed using different divalent metals at a final concentration of 5 mM: ZnCl_2_, FeCl_3_·6H_2_O, CoCl_2_·6H_2_O, CuCl_2_·2H_2_O, MnCl_2_, MgCl_2_·6H_2_O, CaCl_2_·2H_2_O, and FeSO_4_·7H_2_O. In all cases, 0.5 µM EDTA was used to avoid possible interferences. As potential electron acceptors, in addition to INT, cytochrome *c*, potassium ferricyanide, and DCPIP were used.

##### Physiological Reconstitution of D-Malate Oxidase Activity

To determine if D-malate oxidase transfers electrons from D-malate to the electron transport chain, the following assay was designed: using a Clark-type oxygen electrode (Hansatech), oxygen consumption was observed in a reaction mixture containing 100 μL of 0.5 M buffer pH 9, 10 μL of 200 mM ZnCl_2_, 20 µL of membrane fraction cell extract, 25 μL of 200 mM D-malate, and 745 µL of water. The reaction was initiated by adding 100 µL of concentrated periplasmic fraction cell extract (concentrated by 40/60 salt precipitation).

##### Determination of the Reaction Product of D-Malate Oxidase

To determine oxaloacetate (OAA) as the potential product of the oxidation reaction of D-malate catalyzed by DMCO, an experiment coupled with malate dehydrogenase (MDH) was designed, measuring the disappearance of NADH. In a final volume of 100 μL, 50 mM Tris/HCl pH 8, 200 μM INT, 2 mM D-malate, and periplasmic fraction extract from *Ectopseudomonas* partially purified by FPLC chromatography on a Mono-Q 5/50 GL column (GE Healthcare, Chicago, IL, USA) installed in a fast protein liquid chromatography system (Äkta purifier, GE Healthcare, Chicago, Illinois, USA) were added. The mixture was created with distilled water and incubated at 50 °C for 15 min. Then, 100 mM Tris/HCl pH 8 and distilled water were added to a final volume of 500 µL. The kinetics of this reaction mixture were measured at 340 nm. After more than one minute, 50 µL of 2 mM NADH (final concentration 200 μM) and 1 µL of commercial malate dehydrogenase (MDH) (L-malate dehydrogenase from pig heart, approximately 1200 units/mg protein, F. Hoffmann-La Roche Ltd., Basel, Switzerland) were added, and the disappearance of NADH was recorded.

### 4.7. DNA Manipulation Techniques

#### 4.7.1. Extraction and Purification of Nucleic Acids

For the extraction and purification of genomic DNA from *Ectopseudomonas oleovorans* R1D-DMCO^−^, the commercial kit “GeneJET Genomic DNA Purification Kit” (Thermo Fisher Scientific Inc., Massachusetts, USA) was used following the manufacturer’s instructions. After extraction, the concentration was checked by fluorimetry using the Qubit 4 Fluorometer (Thermo Fisher Scientific Inc., Massachusetts, USA) with the “Qubit 1X dsDNA BR Assay kit”. The DNA was stored at −20 °C for subsequent use. For the extraction and purification of plasmid DNA from both *Ectopseudomonas oleovorans* and *E. coli* XL1-Blue, the commercial kit “Fast DNA-spin™ Plasmid DNA Purification Kit” (Intron Biotechnology, Seongnam-si, Gyeonggi-do, Republic of Korea) was used following the manufacturer’s instructions. After extraction, the concentration was checked by fluorimetry using the Qubit 4 Fluorometer with the “Qubit 1X dsDNA BR Assay kit”. The DNA was stored at −20 °C for subsequent use.

#### 4.7.2. Preparation of Electrocompetent Cells

Electrocompetent cells were prepared to introduce foreign DNA into living cells. This protocol was carried out under sterile conditions prior to electroporation and was performed for both *E. coli* XL1-Blue and *Ectopseudomonas oleovorans* R1D. The protocol started with the preparation of a 50 mL LB preculture at pH 7.5 for *E. coli* XL1-Blue and pH 8.5 for CECT 5344. For *Ectopseudomonas oleovorans* R1D, 50 µL of rifampicin was added. *E. coli* XL1-Blue and *Ectopseudomonas oleovorans* R1D were incubated overnight at 37 °C and 30 °C, respectively. The following day, two 200 mL flasks of LB at pH 7.5 or pH 8.5 depending on the strain were inoculated, prewarmed to the optimal temperature for each strain, with a 1:50 dilution. These media did not contain antibiotics. They were incubated in an Incubator 1000 at the corresponding temperature until an OD_600_ nm ≈ 0.3–0.4 is reached, measured with a spectrophotometer (Heλios, Thermo Fisher Scientific Inc., Massachusetts, USA). Once this optical density was achieved, the flasks were placed on ice and we kept all subsequent steps cold. The contents of the flasks were distributed into 50 mL tubes, 25 mL per tube. It was centrifugated for 10 min at 4000 rpm at 4 °C in a Centrifuge 5810R (Eppendorf, Hamburg, Germany). The supernatant was discarded, and the pellet was resuspended in 10 mL of pre-chilled 0.3 M sucrose. It was centrifugated again for 10 min at 4000 rpm at 4 °C, and we discarded the supernatant, and resuspended it in 1 mL of pre-chilled 0.3 M sucrose. The volume was transferred into a 1.5 mL tube and centrifugated for 2 min at 8000 rpm at 4 °C in a Centrifuge 5804 R (Eppendorf). The supernatant was discarded and resuspended in 500 µL of 0.3 M sucrose. The 100 µL portions were aliquoted and stored at −80 °C until use.

#### 4.7.3. Transformation of Electrocompetent Cells

Transformation by electroporation was performed by adding 100 µL of electrocompetent cells (acting as the vector) and the DNA to be inserted into a Gene Pulser Cuvette (BioRad, California, USA). Once the vector and insert DNA are in the cuvette, a pulse is applied using a MicroPulser electroporator (BioRad, Hercules, CA, USA). After the pulse, 1 mL of LB medium (pH depending on the strain as mentioned earlier) is added, and the entire volume is collected into a 1.5 mL tube. For the transformation of *E. coli* XL1-Blue, 5 µL of the ligation of pBKS^+^-DMCO^+^-Gm^R^ DNA was used as the insert. For *P. pseudoalcaligenes* R1D, 10 µL of plasmid DNA from the previous transformation of *E. coli* XL1-Blue with pBKS^+^-DMCO^+^-Gm^R^ was used. The transformation tube was incubated at 30 °C for *Ectopseudomonas oleovorans* for approximately 2–3 h or at 37 °C for 1 h for *E. coli*. After incubation, for *E. coli* XL1-Blue, the contents were spread evenly on LB-Agar plates, pH 7.5, with gentamicin using a sterile L-shaped spreader. The plates were labeled and incubated at 37 °C for 24–48 h. For *Ectopseudomonas oleovorans* R1D, the same procedure was followed but on LB-Agar, pH 8.5, with rifampicin and gentamicin, incubated at 30 °C for 48–72 h.

#### 4.7.4. DNA Amplification by Polymerase Chain Reaction (PCR)

The oligonucleotide sequences used as primers in PCRs were BN5_4044F: CTTCTCGCCGATCATCCACT and BN5_4044R: GCACCGGGTTGTTCAGTTCG. PCR conditions were as follows: initial denaturation at 95 °C for 1–3 min, followed by 25 cycles of 30 s denaturation at 95 °C, 30 s annealing at 61 °C, and 1 min extension at 72 °C. A final extension step of 5 min was added.

## Figures and Tables

**Table 1:** Distribution of enzymatic activities after the subcellular fractionation. Following the subcellular fractionation, the indicated enzymatic activities were measured as described in the Section 4. Red values indicate the subcellular fraction in which the percentage of the specific activity (S.A.) for each enzyme was highest. The data shown are from a representative experiment conducted in triplicate, with similar results.

	Aconitase		Nitrate Reductase		Succinate Oxidase		DMCO	
	S.A. (U/mg)	% S.A.	S.A. (U/mg)	% S.A.	S.A. (U/mg)	% S.A.	S.A. (U/mg)	% S.A.
Periplasm	9.40 × 10^8^	20.8	4.81 × 10^5^	97.8	0.00	0.00	82.83	83.6
Cytoplasm	3.52 × 10^9^	77.8	9.73 × 10^3^	1.98	0.00	0.00	14.32	14.4
Membranes	6.13 × 10^7^	1.3	0.00	0.00	2.18 × 10^5^	100.00	1.85	1.8
	**ACONITASE**		**NITRATE REDUCTASE**		**SUCCINATE OXIDASE**		**DMC** **O**	
	**S.A. (U/mg)**	**% S.A.**	**S.A. (U/mg)**	**% S.A.**	**S.A. (U/mg)**	**% S.A.**	**S.A. (U/mg)**	**% S.A.**
Periplasm	9.40 × 10^8^	20.8	4.81 × 10^5^	97.8	0.00	0.00	82.83	83.6
Cytoplasm	3.52 × 10^9^	77.8	9.73 × 10^3^	1.98	0.00	0.00	14.32	14.4
Membranes	6.13 × 10^7^	1.3	0.00	0.00	2.18 × 10^5^	100.00	1.85	1.8

## Data Availability

The original contributions presented in this study are included in the article. Further inquiries can be directed to the corresponding author(s).

## Abbreviations

The following abbreviations are used in this manuscript:

2DHG	D-2-hydroxyglutarate
2DHGDH	D-2-hydroxyglutarate dehydrogenase
3-PHP	3-phosphohydroxypyruvate
D3PG	D-3-phosphoglycerate
DMCO	D-malate:cytochrome *c* oxidoreductase
ETC	Electron Transport Chain
aKG	2-Ketoglutarate acid
MDH	L-malate dehydrogenase
MQO	L-malate:quinone oxidoreductase
PAGE	Polyacrylamide Gel Electrophoresis
TCA	Tricarboxylic Acid Cycle
